# Knowledge, attitudes, and practices among patients with diabetes mellitus and hyperuricemia toward disease self-management

**DOI:** 10.3389/fpubh.2024.1426259

**Published:** 2024-09-27

**Authors:** Dan Wang, Zhixin Liu, Yu Liu, Lingfei Zhao, Lijuan Xu, Shanshan He, Binhong Duan

**Affiliations:** ^1^Department of Endocrinology, Heilongjiang Provincial Hospital, Harbin, China; ^2^The First Clinical College of Medicine, Mudanjiang Medical University, Mudanjiang, China

**Keywords:** knowledge, attitudes, practices, diabetes mellitus, hyperuricemia, disease management, cross-sectional study

## Abstract

**Background:**

This study aimed to assess the knowledge, attitudes and practices (KAP) among patients with diabetes mellitus and hyperuricemia toward disease self-management.

**Methods:**

This web-based cross-sectional study was conducted between June 2023 and January 2024 at Heilongjiang Provincial Hospital. A self-designed questionnaire was developed to collect demographic information of patients with diabetes mellitus and hyperuricemia, and assess their knowledge, attitudes and practices toward disease self-management.

**Results:**

A total of 482 participants were enrolled in this study, among them, 364 (75.52%) were male, 235 (48.76%) were aged between 40 and 59 years, 226 (46.89%) had a body mass index (BMI) ranging from 24 to 28 kg/m^2^, 337 (69.92%) had received a diagnosis of diabetes for a duration of 2 years or more, while 245 (50.83%) had been diagnosed with hyperuricemia for a similar duration. Their median (range) knowledge, attitude and practice scores were 10.00 (9.00, 11.00) (possible range: 0–12), 38.00 (36.00, 40.00) (possible range: 9–45), and 30.00 (26.00, 34.75) (possible range: 10–50), respectively. The path analysis demonstrated that knowledge had direct effects on attitude (*β* = 0.508, *p* < 0.001), and attitude had direct effects on practice (*β* = 0.448, *p* < 0.001). Additionally, there was an indirect effect of knowledge on practice mediated through attitude, with a path coefficient of 0.228 (*p* < 0.001).

**Conclusion:**

This study demonstrates that patients with diabetes mellitus and hyperuricemia exhibit relatively proficient responses to certain items within the KAP dimensions. However, it also exposes a certain degree of inadequacy in the KAP level toward disease management. Interventions should focus on improving patients’ understanding of their conditions while fostering positive attitudes, ultimately translating into better self-management practices.

## Introduction

The increasing prevalence of metabolic diseases, such as diabetes mellitus and hyperuricemia, has become a significant public health issue worldwide, underscoring the importance of addressing these conditions to improve global health outcomes (1). Hyperuricemia, characterized by elevated serum uric acid levels, is closely linked with metabolic disorders, including type 2 diabetes and metabolic syndrome, highlighting a complex interrelationship that contributes to the burden of metabolic diseases (2). Moreover, approximately 25% of individuals with diabetes are affected by hyperuricemia, indicating a substantial overlap between these conditions and emphasizing the need for integrated management strategies (3).

Management of diabetes mellitus and hyperuricemia requires a comprehensive approach, emphasizing the role of self-management in controlling these conditions. Dietary factors, such as the intake of alcohol, red meat, and seafood, are established risk factors for hyperuricemia and gout flares, suggesting that dietary modifications can significantly reduce the risk of these complications (4). Additionally, physical activity, weight loss, and a healthy diet are crucial for preventing diabetes and its complications, underscoring the importance of lifestyle interventions in managing these metabolic diseases and improving patient outcomes (5). Effective self-management strategies, including lifestyle and dietary modifications, are essential for mitigating the impact of these conditions and enhancing the quality of life for affected individuals.

The Knowledge-Attitude-Practice (KAP) model plays a crucial role in understanding and influencing health behaviors, serving as a fundamental framework within health literacy research. This model posits that knowledge positively affects attitudes, which in turn, guide individual practices (6). Employing the KAP theory is particularly relevant for populations dealing with complex, interrelated health issues such as diabetes mellitus and hyperuricemia. Despite the existence of KAP studies on diabetes and hyperuricemia conducted separately, there is a deficiency in research investigating the KAP pertaining to self-management among patients with both conditions (7–9). This gap highlights the need to assess the KAP among patients with diabetes mellitus and hyperuricemia toward disease self-management.

## Materials and methods

### Study design and participants

This cross-sectional study was conducted between June 2023 and January 2024 at Heilongjiang Provincial Hospital. The study was ethically approved by the Ethics Committee of Heilongjiang Provincial Hospital (Approval No. (2023)075). This study used convenience sampling to recruit the patients with diabetes mellitus and hyperuricemia and the inclusion criteria and exclusion criteria were as follows:

The inclusion criteria for this study were as follows: (1) aged 18 years and older, (2) diagnosed with both diabetes mellitus and hyperuricemia, supported by relevant diagnostic reports or medical records, (3) confirmation of informed consent on the first page of the questionnaire.

The exclusion criteria were as follows: (1) those who self-reported their participation in a similar study, and (2) those who did not pass the validation of the trap questions (K7 & K14, refer to the *Questionnaire* section).

### Questionnaire

The questionnaire was developed in accordance with China multi-disciplinary expert consensus on diagnosis and treatment of hyperuricemia and related diseases (10), Comprehensive Medical Evaluation and Assessment of Comorbidities: Standards of Care in Diabetes-2023 (11) and relevant literature on disease management (12–14). The initial draft underwent refinement based on feedback from three senior experts, comprising two endocrinologists and one rheumatologist. Following this, a pilot study was conducted on a limited scale (*n* = 24), resulting in a Cronbach’s alpha coefficient value of 0.788, indicating good internal consistency.

The final questionnaire was in Chinese and encompassed four dimensions: demographic information, knowledge, attitudes, and practices. The demographic section consisted of 15 items, while the knowledge, attitude, and practice dimensions included 14, 9, and 10 items, respectively.

Certain questions were designed as “trap questions” to maintain the questionnaire’s quality within the study: Question 11 in the demographic section, Questions K7 and K14 in the knowledge dimension. For instance, selecting option “e” alongside any other option for Question 11 led to the exclusion of the corresponding questionnaire from analysis. Likewise, responses to K7 and K14, which conveyed contrasting meanings, were scrutinized. Patients who selected both “correct” and “incorrect” for these questions were recognized as encountering logical conflicts and thus, were omitted from the survey. Consequently, knowledge items were scored 1 point for correct answers and 0 points for incorrect ones, yielding a possible score range of 0–12. Attitude and practice items were rated on a five-point Likert scale, ranging from very positive (5 points) to very negative (1 point) for attitude, and very consistent (5 points) to very inconsistent (1 point) for practice, with respective score ranges of 9–45 and 10–50.

The sample size calculation was based on the guideline that it should be 5 to 10 times the number of items (15). With 28 items in the KAP questionnaire, the required sample size was calculated to be at least 280 participants. Accounting for an anticipated 20% of invalid responses, the adjusted minimum required sample size was 336 participants. This ensures that the study has sufficient statistical power and reliability for analysis.

Data collection was facilitated through an online questionnaire hosted on Sojump.^1^ Participants were prompted to begin the electronic questionnaire by selecting the option “I agree to participate in this study” prior to responding to the questions. All data collection procedures were conducted anonymously. To prevent duplicate submissions, IP restrictions were enforced, restricting completion of the survey to a single instance per IP address.

### Statistical analysis

Statistical analyses were conducted using STATA 17.0 (Stata Corporation, College Station, TX, USA). Continuous variables were described as medians (25th percentile, 75th percentile) or means (SD) depending on the normality of the data, which was assessed using the Shapiro–Wilk test. Between-group comparisons were performed using the Wilcoxon rank-sum test or the Kruskal-Wallis analysis of variance, as appropriate. Categorical variables were presented as N (%). Spearman correlation analysis was employed to assess the relationships between knowledge, attitude, and practice scores. In the multivariate analysis, the median score (for non-normally distributed scores, such as knowledge and attitude scores) or the mean score (for normally distributed scores, such as practice scores) was used as the cut-off value. Thresholds for knowledge, attitudes, and practices were set at 10, 38, and 30 points, respectively. Variables with *p* < 0.05 in univariate analysis were included in the multivariate regression. Path analysis of the relationships among knowledge, attitudes, and practices in patients with diabetes mellitus and hyperuricemia regarding disease management was conducted using AMOS 24.0 (IBM, NY, USA). The path analysis tested the following primary hypotheses: (1) Knowledge has direct effects on attitudes; (2) Knowledge has direct effects on practices; and (3) Attitudes have direct effects on practices. A two-sided *p*-value <0.05 was considered statistically significant in this study.

## Results

Initially, 531 questionnaires were collected. Of these, 49 questionnaires were excluded due to incorrect answers to the trap questions. Consequently, a total of 482 questionnaires were ultimately included in the study. The overall validity rate of the questionnaires was 90.77%.

A total of 482 participants were included in this study. Among them, 364 (75.52%) were male, 235 (48.76%) were aged between 40 and 59 years, 226 (46.89%) had a body mass index (BMI) ranging from 24 to 28 kg/m^2^, and 194 (40.25%) had attained education up to high school or technical school level. Moreover, 337 (69.92%) had received a diagnosis of diabetes for a duration of 2 years or more, while 245 (50.83%) had been diagnosed with hyperuricemia for a similar duration. Furthermore, 177 (36.72%) were smokers, 241 (50%) were consumers of alcohol, and 397 (82.37%) exhibited a preference for diets rich in heavy oils, salt, or other stimulating ingredients. Additionally, 320 (66.39%) did not engage in regular exercise (Table 1).

**Table 1:** Demographic characteristics and scores in knowledge, attitudes, and practices.

		Knowledge		Attitude		Practice	
*N* = 482	*N* (%)	Median [25, 75%] or mean (SD)		Median [25, 75%] or mean (SD)		Median [25, 75%] or mean (SD)	
Total score	482 (100)	10.00 [9.00, 11.00]		38.00 [36.00, 40.00]		30.00 [26.00, 34.75]	
Gender			<0.001		0.713		0.114
Male	364 (75.52)	10.00 [9.00, 11.00]		38.00 [36.00, 40.00]		30.37 (6.10)	
Female	118 (24.48)	10.00 [8.00, 11.00]		38.00 [36.00, 39.75]		31.40 (6.13)	
Age (years)			<0.001		0.002		0.188
18–20	6 (1.24)	10.50 [10.00, 11.00]		39.00 [36.50, 40.00]		35.50 [33.25, 37.75]	
21–39	65 (13.49)	11.00 [9.00, 12.00]		39.00 [37.00, 40.00]		29.00 [26.00, 34.00]	
40–59	235 (48.76)	11.00 [10.00, 11.00]		38.00 [36.00, 40.00]		30.00 [26.00, 33.00]	
≥60	176 (36.51)	10.00 [8.00, 11.00]		37.00 [35.00, 39.00]		31.00 [27.00, 36.00]	
BMI (kg/m^2^)			0.008		0.004		0.343
<18.5	2 (0.41)						
18.5 (inclusive)-24	103 (21.37)	10.00 [9.00, 11.00]		37.00 [35.00, 39.00]		31.00 [27.00, 35.00]	
24 (inclusive)-28	226 (46.89)	10.00 [9.00, 11.00]		38.00 [36.00, 40.00]		30.00 [27.00, 34.00]	
≥28	151 (31.33)	10.00 [9.00, 11.00]		38.00 [36.00, 40.00]		30.00 [25.00, 34.00]	
Residence			<0.001		0.007		0.023
Rural	62 (12.86)	9.00 [8.00, 10.00]		37.00 [36.00, 39.00]		28.98 (6.69)	
Urban	420 (87.14)	10.00 [9.00, 11.00]		38.00 [36.00, 40.00]		30.87 (6.00)	
Marital status			0.003		0.004		0.574
Single	38 (7.88)	10.00 [10.00, 11.00]		38.00 [35.25, 40.00]		29.87 (6.98)	
Married	421 (87.34)	10.00 [9.00, 11.00]		38.00 [36.00, 40.00]		30.76 (6.02)	
Divorced	9 (1.87)	11.00 [10.00, 11.00]		38.00 [36.00, 39.00]		30.22 (6.67)	
Widowed	14 (2.9)	8.50 [7.25, 9.00]		34.50 [33.25, 36.00]		28.86 (6.43)	
Education			<0.001		<0.001		<0.001
Junior high school and below	140 (29.05)	9.00 [8.00, 10.00]		36.00 [34.00, 38.00]		28.61 (6.24)	
High school and technical school	194 (40.25)	10.00 [9.00, 11.00]		38.00 [37.00, 40.00]		30.89 (5.47)	
College and bachelor’s degree	139 (28.84)	11.00 [10.00, 12.00]		39.00 [37.00, 40.00]		32.08 (6.38)	
Master’s degree and above	9 (1.87)	12.00 [11.00, 12.00]		40.00 [38.00, 40.00]		33.67 (5.27)	
Occupation			<0.001		<0.001		0.014
Formal employee/part-time/freelancer	254 (52.7)	11.00 [10.00, 12.00]		39.00 [37.00, 40.00]		30.22 (6.22)	
Unemployed	6 (1.24)	10.00 [8.25, 11.00]		35.00 [34.00, 37.50]		26.33 (8.33)	
Retired	176 (36.51)	10.00 [9.00, 11.00]		38.00 [36.00, 39.00]		31.65 (5.92)	
Other	46 (9.54)	9.00 [7.25, 10.00]		36.00 [34.00, 39.00]		29.52 (5.40)	
Monthly *per capita* income (CNY)			<0.001		<0.001		<0.001
a. < 2000	43 (8.92)	9.00 [7.50, 10.00]		36.00 [33.00, 38.00]		27.93 (5.73)	
b. 2000–5000	181 (37.55)	10.00 [8.00, 10.00]		37.00 [35.00, 39.00]		29.92 (6.13)	
c. 5000–10000	199 (41.29)	11.00 [10.00, 11.00]		39.00 [37.00, 40.00]		30.89 (5.87)	
d. 10000–20000	44 (9.13)	11.00 [11.00, 12.00]		39.00 [37.75, 40.00]		33.41 (5.98)	
e. > 20000	15 (3.11)	11.00 [11.00, 12.00]		38.00 [35.50, 39.50]		35.13 (5.45)	
Time since diabetes diagnosis			0.147		0.074		0.001
Within six months	107 (22.2)	10.00 [8.00, 11.00]		39.00 [36.50, 40.00]		31.85 (6.07)	
More than six months to within one year	22 (4.56)	11.00 [10.00, 12.00]		39.00 [37.00, 40.00]		33.91 (5.99)	
More than one year to within two years	16 (3.32)	10.50 [9.75, 11.00]		38.50 [36.75, 39.00]		32.88 (6.52)	
Two years or more	337 (69.92)	10.00 [9.00, 11.00]		38.00 [36.00, 39.00]		29.91 (5.99)	
Time since hyperuricemia diagnosis			0.004		0.776		0.166
Within six months	199 (41.29)	10.00 [8.50, 11.00]		38.00 [36.00, 39.00]		31.00 [27.00, 35.00]	
More than six months to within one year	19 (3.94)	10.00 [9.00, 11.00]		38.00 [36.50, 39.50]		30.00 [29.50, 37.00]	
More than one year to within two years	19 (3.94)	11.00 [9.50, 11.00]		39.00 [35.50, 40.00]		34.00 [29.50, 36.50]	
Two years or more	245 (50.83)	10.00 [10.00, 11.00]		38.00 [36.00, 40.00]		30.00 [26.00, 34.00]	
Comorbidities (Multiple choices)			/		/		/
Hypertension	291 (60.37)	/		/		/	
Dyslipidemia	345 (71.58)	/		/		/	
Heart disease	134 (27.8)	/		/		/	
Kidney disease	35 (7.26)	/		/		/	
None of the above	49 (10.17)	/		/		/	
Smoking			0.560		0.938		<0.001
Yes	177 (36.72)	10.00 [9.00, 11.00]		38.00 [36.00, 40.00]		29.30 (5.97)	
No	305 (63.28)	10.00 [9.00, 11.00]		38.00 [36.00, 40.00]		31.39 (6.08)	
Alcohol consumption			0.125		0.558		<0.001
Yes	241 (50)	10.00 [9.00, 11.00]		38.00 [36.00, 39.00]		29.41 (5.89)	
No	241 (50)	10.00 [9.00, 11.00]		38.00 [36.00, 40.00]		31.84 (6.10)	
Preference for stimulating diet			0.704		0.302		<0.001
Yes	397 (82.37)	10.00 [9.00, 11.00]		38.00 [36.00, 40.00]		29.88 (5.92)	
No	85 (17.63)	10.00 [9.00, 11.00]		37.00 [35.00, 40.00]		34.09 (5.86)	
Regular exercise			<0.001		<0.001		<0.001
Yes	162 (33.61)	11.00 [10.00, 11.00]		39.00 [37.00, 40.00]		35.15 (5.15)	
No	320 (66.39)	10.00 [9.00, 11.00]		38.00 [35.00, 39.00]		28.33 (5.23)	

The three knowledge items with the highest correctness rates were as follows: “Reasonable exercise can increase energy expenditure, help control body weight, and reduce blood uric acid and blood glucose levels.” (K11) with a correctness rate of 98.76%, “The occurrence of diabetes and hyperuricemia may be related to changes in dietary structure, such as increased intake of sugars and fats, and insufficient physical activity.” (K2) with a correctness rate of 97.72%, “The main complications of diabetes include retinopathy, diabetic nephropathy, and diabetic foot, among others.” (K4) with a correctness rate of 97.51%. The three items with the lowest correctness rates were as follows: “Hyperuricemia patients are not necessarily gout patients.” (K6) with a correctness rate of 37.34%, “Hyperuricemia is closely related to hypertension, kidney damage, and cardiovascular disease.” (K8) with a correctness rate of 44.61%, “The incidence of hyperuricemia in diabetes patients far exceeds that in non-individuals with diabetes.” (K7) with a correctness rate of 61.41% (Table 2).

**Table 2:** Knowledge in managing diabetes and hyperuricemia.

	Correctness *N* (%)
K1. Diabetes is a chronic disease caused by various factors, including genetic and environmental ones. (True)	437 (90.66)
K2. The occurrence of diabetes and hyperuricemia may be related to changes in dietary structure, such as increased intake of sugars and fats, and insufficient physical activity. (True)	471 (97.72)
K3. Hyperuricemia is mainly caused by disorders in the human body’s purine metabolism. (True)	334 (69.29)
K4. The main complications of diabetes include retinopathy, diabetic nephropathy, and diabetic foot, among others. (True)	470 (97.51)
K5. The main complications of hyperuricemia include gout, uric acid nephropathy, uric acid kidney stones, and metabolic syndrome. (True)	418 (86.72)
K6. Hyperuricemia patients are not necessarily gout patients. (False)	180 (37.34)
K7. The incidence of hyperuricemia in diabetes patients far exceeds that in non-individuals with diabetes. (True)	296 (61.41)
K8. Hyperuricemia is closely related to hypertension, kidney damage, and cardiovascular disease. (True)	215 (44.61)
K9. Diabetes patients with concomitant hyperuricemia should limit sugar intake but do not need to restrict alcohol or high purine (such as shellfish and animal offal) intake. (False)	462 (95.85)
K10. Drinking alcohol may further induce hyperuricemia and may exacerbate insulin resistance and worsen the progression of diabetes. Therefore, diabetes patients with concomitant hyperuricemia should avoid alcohol consumption as much as possible. (True)	470 (97.51)
K11. Reasonable exercise can increase energy expenditure, help control body weight, and reduce blood uric acid and blood glucose levels. (True)	476 (98.76)
K12. Supplementing water can dilute urine, facilitate uric acid excretion, and improve the condition of hyperuricemia. (True)	437 (90.66)
K13. Diabetes patients with concomitant hyperuricemia need to receive treatment for both conditions simultaneously. (True)	456 (94.61)
K14. The incidence of hyperuricemia in diabetes patients is not the same as that in non-individuals with diabetes. (False)	296 (61.41)

Among the participants, 82.78% strongly agreed that their condition could be improved through active disease management (A9), 77.59% strongly agreed that it was very important to implement disease self-management (A3), and 71.58% had very full trust in the treatment plan that the doctor had developed for them (A5). Concurrently, 58.09% were willing to learn about disease self-management through various forms (A1). However, nearly half of the participants (49.38%) reported experiencing anxiety or concern regarding potential complications linked to diabetes or hyperuricemia. These emotional states possess the potential to influence their quality of life and adherence to treatment protocols (A8) (Table 3).

**Table 3:** Attitudes in managing diabetes and hyperuricemia.

	Strongly agree *N* (%)	Agree *N* (%)	Neutral *N* (%)	Disagree *N* (%)	Strongly disagree *N* (%)
A1. You are willing to learn about disease self-management through books, the internet, short videos, consulting specialized doctors, and other forms. (Positive)	106 (21.99)	280 (58.09)	59 (12.24)	35 (7.26)	2 (0.41)
A2. You are happy to exchange experiences in disease prevention and control with other patients. (Positive)	228 (47.3)	186 (38.59)	28 (5.81)	40 (8.3)	0 (0)
A3. You believe that implementing disease self-management is extremely important. (Positive)	374 (77.59)	103 (21.37)	4 (0.83)	0 (0)	1 (0.21)
A4. You believe that your lifestyle (such as smoking, drinking, diet) has a significant impact on the occurrence or exacerbation of hyperuricemia and diabetes. (Positive)	332 (68.88)	133 (27.59)	11 (2.28)	5 (1.04)	1 (0.21)
A5. You have full trust in the treatment plans developed by your doctor. (Positive)	345 (71.58)	122 (25.31)	14 (2.9)	1 (0.21)	0 (0)
A6. You acknowledge the importance of regular check-ups for diabetes patients with concomitant hyperuricemia. (Positive)	259 (53.73)	197 (40.87)	22 (4.56)	4 (0.83)	0 (0)
A7. You acknowledge that diabetes patients with concomitant hyperuricemia should remain vigilant about the symptoms related to the disease and its complications. (Positive)	230 (47.72)	201 (41.7)	24 (4.98)	25 (5.19)	2 (0.41)
A8. You feel anxious or worried about the potential complications of diabetes or hyperuricemia. (Negative)	238 (49.38)	183 (37.97)	29 (6.02)	30 (6.22)	2 (0.41)
A9. You believe that active disease management can improve the condition. (Positive)	399 (82.78)	75 (15.56)	6 (1.24)	2 (0.41)	0 (0)

Patients’ activity or frequency of performing these practices items varied greatly, with 54.77% strictly adhering to the prescribed medication and dosage for disease self-management (P4), and 52.9% communicating closely with their physicians to make timely adjustments to their self-management plan (P10). Strikingly, 51.24% never participated in exercise to improve their condition (P5). Intriguingly, 31.95% often consciously communicate with other patients about their condition and disease self-management in various ways in their lives (P1), while a similar proportion (37.14%) rarely do so (Table 4).

**Table 4:** Practices in managing diabetes and hyperuricemia.

	Very consistent *N* (%)	Quite consistent *N* (%)	Consistent *N* (%)	Quite inconsistent *N* (%)	Very inconsistent *N* (%)
P1. You consciously communicate about your condition and disease self-management knowledge with other diabetes patients with concomitant hyperuricemia through the internet, WeChat groups, chatting with family and friends, etc.	12 (2.49)	154 (31.95)	76 (15.77)	179 (37.14)	61 (12.66)
P2. You regularly visit the hospital for follow-up appointments to check your condition and screen for potential complications.	20 (4.15)	78 (16.18)	101 (20.95)	171 (35.48)	112 (23.24)
P3. During the disease self-management process, you regularly monitor your blood glucose and uric acid levels.	46 (9.54)	92 (19.09)	92 (19.09)	164 (34.02)	88 (18.26)
P4. During the disease self-management process, you strictly adhere to the medication usage methods and dosages prescribed by your doctor.	264 (54.77)	165 (34.23)	32 (6.64)	13 (2.7)	8 (1.66)
P6. You maintain a balanced diet (controlling sugar and fat intake, increasing vitamin intake, etc.) to control the conditions of diabetes and hyperuricemia.	13 (2.7)	75 (15.56)	100 (20.75)	201 (41.7)	93 (19.29)
P7. During the disease self-management process, you try to avoid smoking, alcohol, and other factors that may exacerbate diabetes and hyperuricemia.	61 (12.66)	138 (28.63)	63 (13.07)	171 (35.48)	49 (10.17)
P8. During the disease self-management process, you remain vigilant about the related complications of diabetes and hyperuricemia, and if you notice any related symptoms, you seek medical attention immediately.	20 (4.15)	101 (20.95)	135 (28.01)	205 (42.53)	21 (4.36)
P9. When you notice that the disease is negatively affecting your mental health, you actively communicate with family and friends or seek professional psychological therapy.	54 (11.2)	256 (53.11)	58 (12.03)	93 (19.29)	21 (4.36)
P10. You communicate with your doctor about the implementation of self-management for diabetes and hyperuricemia, and you adjust the management plan promptly.	255 (52.9)	181 (37.55)	22 (4.56)	21 (4.36)	3 (0.62)
	a. Never participate in exercise *N* (%)	b. Occasionally participate in exercise (3 times or less per month) *N* (%)	c. Engage in exercise once or twice a week *N* (%)	d. Engage in exercise three to four times a week *N* (%)	e. Engage in exercise five times or more per week *N* (%)
P5. To improve your condition, how often do you engage in regular exercise?	247 (51.24)	61 (12.66)	16 (3.32)	60 (12.45)	98 (20.33)

Spearman’s analysis was performed to assess the relationship between knowledge, attitudes, and practices. It was shown that the knowledge and the attitudes were positively correlated (*r* = 0.330, *p* < 0.001), and knowledge and practices were also positively correlated (*r* = 0.266, *p* < 0.001). Additionally, there was a positive correlation between attitude and practice scores (*r* = 0.345, *p* < 0.001) (Table 5).

**Table 5:** Spearman correlation analysis among knowledge, attitudes, and practices.

	Knowledge	Attitudes	Practices
Knowledge	1.000		
Attitudes	0.330 (*p* < 0.001)	1.000	
Practices	0.266 (*p* < 0.001)	0.345 (*p* < 0.001)	1.000

Using the median or means scores for each dimension as a cut-off value, the proportions of participants who scored above the cut-off value for knowledge, attitude and practice was 328 (68.05%), 282 (58.51%), and 263 (54.56%) (Table 6).

**Table 6:** Distribution of sufficient knowledge, favorable attitudes, and proactive practices.

Cutoff values: Median for knowledge and attitude; Mean for practice	*N*
Knowledge total score	
Ksum ≥ 10	328 (68.05)
Ksum <= 9	154 (31.95)
Attitude total score	
Asum ≥ 38	282 (58.51)
Asum <= 37	200 (41.49)
Practice total score	
Psum ≥ 32	263 (54.56)
Psum <= 31	219 (45.43)

Multivariate analysis revealed that living in urban areas (OR = 2.58, 95% CI: 1.81–3.68, *p* = 0.007), having a high school or technical school education (OR = 2.02, 95% CI: 1.52–2.69, *p* = 0.014), holding a college or bachelor’s degree (OR = 4.06, 95% CI: 2.78–5.93, *p* < 0.001), being retired (OR = 0.54, 95% CI: 0.41–0.71, *p* = 0.024), having a monthly *per capita* income of 10000–20000 Yuan (OR = 16.94, 95% CI: 5.36–53.53, *p* = 0.014), being diagnosed with hyperuricemia for 2 years or more (OR = 2.18, 95% CI: 1.7–2.78, *p* = 0.001), and not having regular exercise habits (OR = 0.57, 95% CI: 0.44–0.74, *p* = 0.032) were independently associated with knowledge (Table 7). Additionally, knowledge score (OR = 1.26, 95% CI: 1.17–1.36, *p* = 0.003), having a BMI not less than 28 kg/m2 (OR = 2.03, 95% CI: 1.51–2.72, *p* = 0.017), high school or technical school education (OR = 1.84, 95% CI: 1.39–2.43, *p* = 0.030), and not having regular exercise habits (OR = 0.51, 95% CI: 0.41–0.65, *p* = 0.004) were independently associated with attitude (Table 8). Furthermore, knowledge score (OR = 1.24, 95% CI: 1.13–1.36, *p* = 0.020), attitude score (OR = 1.20, 95% CI: 1.14–1.26, *p* = 0.000), being diagnosed with diabetes for 2 years or more (OR = 0.33, 95% CI: 0.24–0.44, *p* < 0.001), not drinking alcohol (OR = 2.22, 95% CI: 1.71–2.9, *p* = 0.002), having no preference for heavy oils, salty, or other stimulating foods (OR = 4.1, 95% CI: 2.89–5.82, *p* < 0.001), and not having regular exercise habits (OR = 0.1, 95% CI: 0.07–0.13, *p* < 0.001) were independently associated with practice (Table 9).

**Table 7:** Univariate and multivariate analysis of factors associated with knowledge.

Knowledge	Univariate analysis		Multivariate analysis	
	OR (95%CI)	*p*	OR (95%CI)	*p*
Gender				
Male				
Female	0.52 (0.34, 0.8)	0.003	1.31 (0.71, 2.42)	0.380
Age (years)				
18–20				
21–39	0.56 (0.03, 3.84)	0.613		
40–59	0.69 (0.04, 4.38)	0.734		
≥60	0.22 (0.01, 1.43)	0.176		
BMI (kg/m^2^)				
<=24				
24 (inclusive)-28	1.71 (1.06, 2.76)	0.029	1.13 (0.63, 2.02)	0.692
≥28	2.08 (1.23, 3.55)	0.007	1.45 (0.76, 2.76)	0.258
Residence				
Rural				
Urban	5.3 (3.04, 9.52)	<0.001	2.58 (1.29, 5.16)	0.007
Marital status				
Single				
Married	0.68 (0.3, 1.42)	0.329	0.92 (0.34, 2.46)	0.870
Divorced	1.09 (0.21, 8.16)	0.926	1.21 (0.16, 9.26)	0.852
Widowed	0.08 (0.02, 0.34)	0.001	0.29 (0.05, 1.62)	0.158
Education				
Junior high school and below				
High school and technical school	4.1 (2.59, 6.56)	<0.001	2.02 (1.16, 3.54)	0.014
College and bachelor’s degree	10.76 (5.98, 20.34)	<0.001	4.06 (1.93, 8.54)	<0.001
Master’s degree and above	12 (2.12, 225.72)	0.021	2.33 (0.16, 33.58)	0.535
Occupation				
Formal employee/part-time/ freelancer				
Unemployed	0.25 (0.05, 1.39)	0.097	1.05 (0.13, 8.52)	0.964
Retired	0.36 (0.24, 0.56)	<0.001	0.54 (0.31, 0.92)	0.024
Other	0.16 (0.08, 0.31)	<0.001	0.58 (0.23, 1.42)	0.231
Monthly *per capita* income (CNY)				
a. < 2000				
b. 2000–5000	2.56 (1.29, 5.29)	0.009	1.53 (0.58, 4.00)	0.389
c. 5000–10000	7.98 (3.94, 16.89)	<0.001	2.39 (0.84, 6.82)	0.102
d. 10000–20000	89.07 (16.7, 1660.17)	<0.001	16.94 (1.78, 161.56)	0.014
e. > 20000	13.46 (3.17, 94.06)	0.002	2.77 (0.38, 20.37)	0.316
Time since diabetes diagnosis				
Within six months				
More than six months to within one year	2.11 (0.77, 6.82)	0.171		
More than one year to within two years	1.86 (0.6, 7.02)	0.308		
Two years or more	1.39 (0.88, 2.18)	0.153		
Time since hyperuricemia diagnosis				
Within six months				
More than six months to within one year	1.23 (0.47, 3.42)	0.681	1.16 (0.35, 3.80)	0.807
More than one year to within two years	2 (0.73, 6.39)	0.199	2.61 (0.72, 9.49)	0.144
Two years or more	2.26 (1.51, 3.4)	<0.001	2.18 (1.35, 3.51)	0.001
Smoking				
Yes				
No	0.79 (0.53, 1.18)	0.261		
Alcohol consumption				
Yes				
No	0.71 (0.48, 1.04)	0.079	0.91 (0.54, 1.53)	0.712
Preference for stimulating diet				
Yes				
No	0.83 (0.51, 1.38)	0.467		
Regular exercise				
Yes				
No	0.51 (0.33, 0.79)	0.002	0.57 (0.34, 0.95)	0.032

**Table 8:** Univariate and multivariate analysis of factors associated with attitudes.

Attitude	Univariate analysis		Multivariate analysis	
	OR (95%CI)	*p*	OR (95%CI)	*p*
Knowledge score	1.46 (1.37, 1.56)	<0.001	1.26 (1.08, 1.47)	0.003
Gender				
Male				
Female	0.91 (0.6, 1.39)	0.661		
Age (years)				
18–20				
21–39	1.41 (0.18, 7.94)	0.705		
40–59	0.78 (0.11, 4.06)	0.774		
≥60	0.49 (0.07, 2.57)	0.415		
BMI (kg/m^2^)				
<=24				
24 (inclusive)-28	2.01 (1.26, 3.23)	0.004	1.61 (0.96, 2.72)	0.073
≥28	2.37 (1.43, 3.97)	0.001	2.03 (1.14, 3.61)	0.017
Residence				
Rural				
Urban	2.16 (1.26, 3.74)	0.005	1.06 (0.54, 2.05)	0.871
Marital status				
Single				
Married	0.86 (0.42, 1.69)	0.67	1.14 (0.52, 2.52)	0.745
Divorced	0.73 (0.17, 3.37)	0.674	0.70 (0.13, 3.65)	0.668
Widowed	0.1 (0.01, 0.42)	0.005	0.40 (0.07, 2.34)	0.306
Education				
Junior high school and below				
High school and technical school	3.02 (1.93, 4.76)	0	1.84 (1.06, 3.18)	0.030
College and bachelor’s degree	3.22 (1.98, 5.29)	0	1.46 (0.75, 2.84)	0.260
Master’s degree and above	12.74 (2.25, 239.7)	0.018	10.07 (0.97, 105.07)	0.053
Occupation				
Formal employee/part-time/freelancer				
Unemployed	0.24 (0.03, 1.27)	0.106	0.23 (0.03, 1.94)	0.177
Retired	0.51 (0.34, 0.75)	0.001	0.77 (0.47, 1.24)	0.274
Other	0.34 (0.18, 0.65)	0.001	0.75 (0.34, 1.67)	0.480
Monthly *per capita* income (CNY)				
a. < 2000				
b. 2000–5000	1.45 (0.74, 2.89)	0.285	0.82 (0.34, 1.98)	0.657
c. 5000–10000	3.3 (1.69, 6.62)	0.001	1.10 (0.42, 2.88)	0.841
d. 10000–20000	4.59 (1.88, 11.85)	0.001	1.01 (0.30, 3.34)	0.988
e. > 20000	1.75 (0.53, 5.87)	0.355	0.31 (0.07, 1.47)	0.141
Time since diabetes diagnosis				
Within six months				
More than six months to within one year	1.28 (0.49, 3.6)	0.622		
More than one year to within two years	0.77 (0.27, 2.3)	0.626		
Two years or more	0.78 (0.5, 1.22)	0.278		
Time since hyperuricemia diagnosis				
Within six months				
More than six months to within one year	1.05 (0.41, 2.81)	0.926		
More than one year to within two years	1.3 (0.5, 3.64)	0.592		
Two years or more	1.12 (0.77, 1.64)	0.551		
Smoking				
Yes				
No	1.02 (0.7, 1.49)	0.915		
Alcohol consumption				
Yes				
No	1.19 (0.83, 1.71)	0.355		
Preference for stimulating diet				
Yes				
No	0.64 (0.4, 1.02)	0.062	0.66 (0.38, 1.13)	0.131
Regular exercise				
Yes				
No	0.51 (0.34, 0.75)	0.001	0.51 (0.33, 0.81)	0.004

**Table 9:** Univariate and multivariate analysis of factors associated with practices.

Practice	Univariate analysis		Multivariate analysis	
	OR (95%CI)	*p*	OR (95%CI)	*p*
Knowledge score	1.34 (1.26, 1.42)	<0.001	1.24 (1.03, 1.49)	0.020
Attitude score	1.27 (1.22, 1.32)	<0.001	1.20 (1.09, 1.33)	0.000
Gender				
Male				
Female	1.42 (0.93, 2.17)	0.106		
Age (years)				
18–20				
21–39	0.19 (0.01, 1.29)	0.144		
40–59	0.24 (0.01, 1.49)	0.19		
≥60	0.26 (0.01, 1.64)	0.219		
BMI (kg/m^2^)				
<=24				
24 (inclusive)-28	0.95 (0.59, 1.51)	0.822		
≥28	0.88 (0.53, 1.45)	0.612		
Residence				
Rural				
Urban	1.79 (1.05, 3.1)	0.034	1.60 (0.77, 3.33)	0.212
Marital status				
Single				
Married	1.23 (0.63, 2.4)	0.545		
Divorced	1.25 (0.29, 5.74)	0.765		
Widowed	1.00 (0.29, 3.46)	1.000		
Education				
Junior high school and below				
High school and technical school	1.84 (1.19, 2.86)	0.007	1.25 (0.67, 2.33)	0.488
College and bachelor’s degree	2.16 (1.34, 3.5)	0.002	1.11 (0.53, 2.33)	0.781
Master’s degree and above	10.98 (1.94, 206.54)	0.026	2.40 (0.21, 26.86)	0.479
Occupation				
Formal employee/part-time/freelancer				
Unemployed	0.47 (0.06, 2.45)	0.388		
Retired	1.36 (0.92, 2)	0.124		
Other	1.22 (0.65, 2.32)	0.537		
Monthly *per capita* income (CNY)				
a. < 2000				
b. 2000–5000	1.28 (0.66, 2.52)	0.473	0.66 (0.27, 1.60)	0.345
c. 5000–10000	1.47 (0.76, 2.88)	0.256	0.50 (0.20, 1.28)	0.163
d. 10000–20000	3.79 (1.56, 9.7)	0.004	0.95 (0.27, 3.34)	0.939
e. >20000	8.21 (1.96, 56.74)	0.010	2.48 (0.36, 17.32)	0.362
Time since diabetes diagnosis				
Within six months				
More than six months to within one year	1.18 (0.46, 3.32)	0.741	0.89 (0.28, 2.87)	0.845
More than one year to within two years	1.65 (0.53, 6.23)	0.412	1.25 (0.27, 5.83)	0.778
Two years or more	0.54 (0.34, 0.84)	0.007	0.33 (0.18, 0.62)	<0.001
Time since hyperuricemia diagnosis				
Within six months				
More than six months to within one year	2.17 (0.8, 6.94)	0.150		
More than one year to within two years	2.17 (0.8, 6.94)	0.150		
Two years or more	0.78 (0.54, 1.14)	0.202		
Smoking				
Yes				
No	1.89 (1.3, 2.75)	0.001	1.38 (0.82, 2.31)	0.222
Alcohol consumption				
Yes				
No	2.22 (1.54, 3.21)	<0.001	2.22 (1.33, 3.70)	0.002
Preference for stimulating diet				
Yes				
No	3.03 (1.81, 5.26)	<0.001	4.10 (2.07, 8.14)	<0.001
Regular exercise				
Yes				
No	0.1 (0.06, 0.17)	<0.001	0.10 (0.05, 0.20)	<0.001

**Figure 1:**
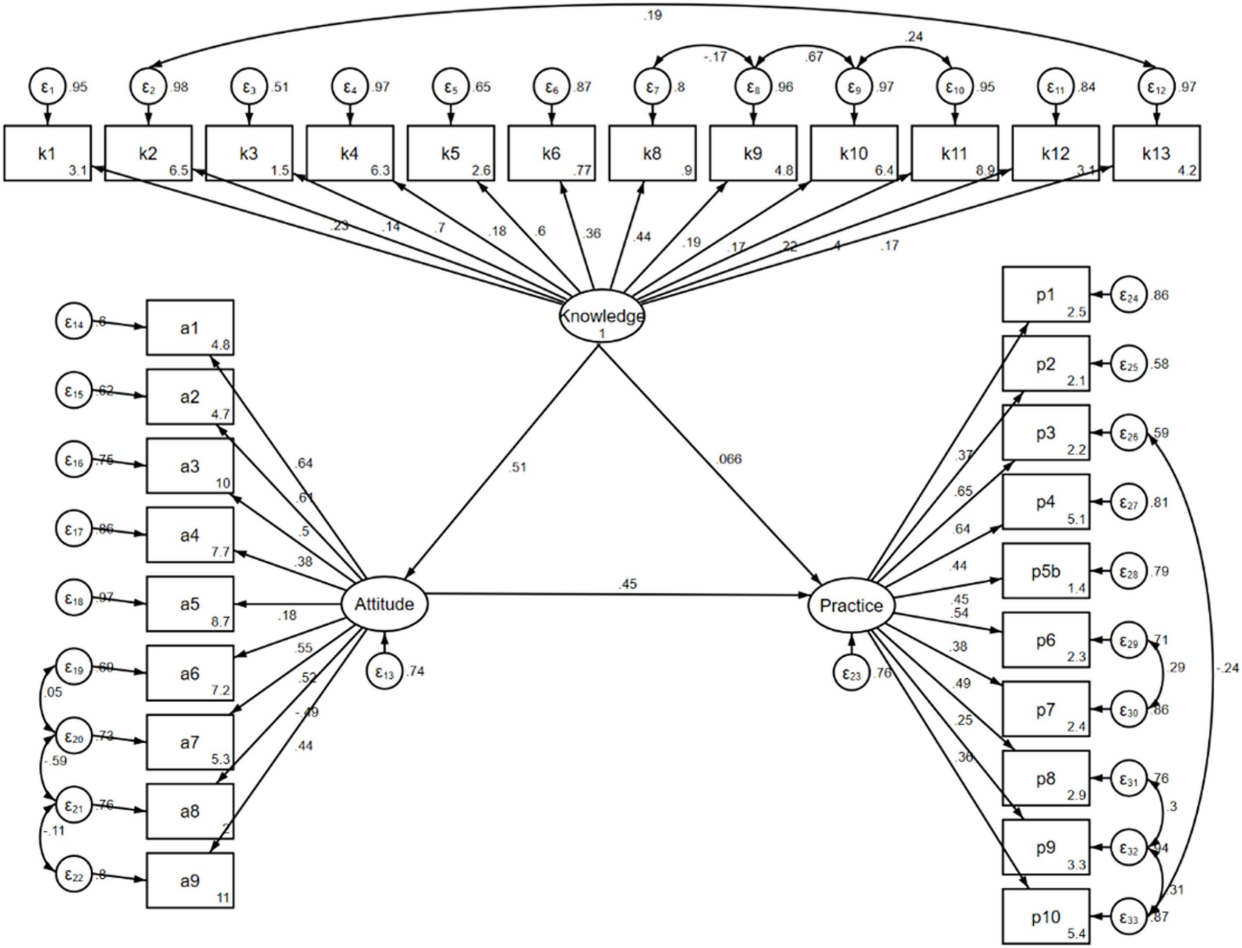
The KAP structural equation model.

The path analysis was established to further investigate whether patients’ knowledge and attitude toward disease self-management affect their practice, whether attitude plays an intermediary role between knowledge and practice, and whether knowledge can directly affect their practice according to the KAP theory. Model fitness indices showed a good model fit (Table 10) and the results demonstrated that knowledge had direct effects on attitude (*β* = 0.508, *p* < 0.001), and attitude had direct effects on practice (*β* = 0.448, *p* < 0.001). Additionally, there was an indirect effect of knowledge on practice mediated through attitude, with a path coefficient of 0.228 (*p* < 0.001) (Tables 11, 12 and Figure 1).

**Table 10:** Model fit indices for the KAP structural equation model.

Indicators	Reference	Results
RMSEA	<0.08 Good	0.050
SRMR	<0.08 Good	0.068
TLI	>0.8 Good	0.812
CFI	>0.8 Good	0.830

**Table 11:** Test results of hypotheses on the relationships among KAP scores.

Indicators		Estimate	*p* > |z|
Asum			
	Ksum	9.33	<0.001
Psum			
	Asum	6.48	<0.001
	Ksum	0.88	0.378

**Table 12:** Total, direct, and indirect effects of KAP scores in the structural equation model.

Model paths		Total effects		Direct effect		Indirect effect	
		*β* (95% CI)	*p*	*β* (95% CI)	*p*	*β* (95% CI)	*p*
Asum							
	Ksum	0.508 (0.453, 0.562)	<0.001	0.508 (0.453, 0.562)	<0.001		
Psum							
	Asum	0.448 (0.360, 0.517)	<0.001	0.448 (0.360, 0.517)	<0.001		
	Ksum	0.293 (0.233, 0.353)	<0.001	0.066 (−0.008, 0.140)	0.378	0.228 (0.183, 0.272)	<0.001

## Discussion

This study demonstrates that patients with diabetes mellitus and hyperuricemia exhibit relatively proficient responses to certain items within the KAP dimensions. However, it also exposes a certain degree of inadequacy in the KAP level toward disease management. Interventions should focus on improving patients’ understanding of their conditions while fostering positive attitudes, ultimately translating into better self-management practices.

The findings from the study revealed important insights into the KAP of patients with diabetes mellitus and hyperuricemia toward disease self-management. Overall, the results indicate that while patients generally possess positive attitudes, their knowledge levels are inadequate, and their practices are suboptimal. Previous research has identified medication management and the conflict between disease and daily life as the primary needs among the people with diabetes in China (16). Given the positive attitudes observed in this study, addressing these needs may contribute to an improvement in knowledge levels and ultimately enhance clinical outcomes for individuals with diabetes with complications.

Several demographic, lifestyle, and health-related characteristics were found to significantly influence KAP scores. For instance, education level, as evidenced by both inter-group comparisons and multivariate analysis, emerges as a significant determinant. In inter-group comparisons, individuals with higher education levels consistently scored higher across domains of health knowledge, attitudes, and practices. This finding aligns with prior research emphasizing the positive impact of education on health literacy and behaviors (17, 18). Moreover, multivariate analysis corroborates the significance of education level as an independent predictor of health knowledge, attitudes, and practices. Another notable factor is the residential area. In inter-group comparisons, urban residents demonstrated higher scores across all domains compared to their rural counterparts, reflecting potential disparities in health literacy and behaviors between urban and rural populations. Multivariate analysis identified residential area as an independent predictor of knowledge scores, indicating its continued relevance even when considering other factors. This may reflect differences in access to health information, lifestyle factors, and healthcare resources between urban and rural settings (19–21). Thus, targeted community education and health promotion initiatives may be instrumental in bridging the urban–rural health gap and improving health knowledge among rural residents.

Moreover, Spearman’s analysis revealed significant positive correlations between knowledge, attitudes, and practices, suggesting that enhancing knowledge may positively influence attitudes and subsequently lead to improved practices. The path analysis further supported these findings, demonstrating direct and indirect effects of knowledge and attitude on practices. These results are consistent with the KAP theory, which posits that enhancing knowledge and fostering positive attitudes are crucial steps toward behavior change (22, 23).

The assessment of participants’ knowledge toward diabetes and hyperuricemia unveiled both strengths and areas for improvement. While participants demonstrated a solid grasp of the relationship between lifestyle factors and disease onset, particularly acknowledging the impact of dietary habits and physical activity, there were notable misconceptions toward the association between hyperuricemia and gout. To address this, healthcare providers could implement targeted educational workshops or seminars focusing on clarifying common misconceptions about hyperuricemia and its potential complications. Additionally, integrating visual aids, such as infographics or educational videos, into patient education materials can enhance understanding and retention of key concepts. By empowering patients with accurate knowledge, healthcare providers can equip them with the necessary tools to make informed decisions about their health and engage effectively in disease self-management practices (24, 25).

Participants exhibited generally positive attitudes toward disease self-management, demonstrating a willingness to engage in proactive health behaviors. However, there was a concerning lack of awareness or denial toward the potential complications of diabetes and hyperuricemia. To address this, healthcare providers could implement targeted psychoeducational interventions aimed at raising awareness about potential complications and providing strategies for coping with anxiety or worry related to disease management (26). Peer support groups or counseling sessions facilitated by trained healthcare professionals can provide opportunities for patients to share experiences, receive support, and learn effective coping strategies. Additionally, incorporating regular discussions about potential complications and coping mechanisms into routine clinic visits can help normalize these conversations and encourage proactive engagement in disease self-management practices.

Analysis of participants’ practices revealed varying levels of consistency in disease management behaviors. While there was a strong adherence to prescribed medication regimens, challenges were observed in maintaining regular exercise habits. Regular physical activity plays a crucial role in disease management and prevention, highlighting the need for tailored interventions to promote exercise adherence. Additionally, participants demonstrated varying degrees of engagement in disease self-management activities such as monitoring blood glucose and uric acid levels and seeking medical attention promptly when necessary. To address this, healthcare providers could work collaboratively with patients to develop personalized exercise plans tailored to their preferences, abilities, and lifestyle constraints. Incorporating enjoyable and accessible physical activities, such as walking groups or chair exercises, can enhance adherence and promote long-term sustainability (27, 28). Furthermore, leveraging technology, such as smartphone apps or wearable activity trackers, can provide real-time feedback and motivation to support exercise adherence. Additionally, implementing regular follow-up appointments with healthcare providers to monitor progress, address challenges, and adjust management plans as needed can provide ongoing support and encouragement for patients to maintain consistent engagement in disease self-management practices (29, 30).

Despite the valuable insights gained from this study, several limitations must be acknowledged. Firstly, the reliance on a web-based survey may introduce selection bias, as it excludes individuals without internet access or those who are less technologically savvy. Additionally, the cross-sectional design of the study limits the ability to establish causality between variables and only provides a snapshot of participants’ KAP at a single point in time. Moreover, the study’s reliance on self-reported data introduces the possibility of recall bias and social desirability bias, potentially affecting the accuracy of responses toward participants’ knowledge, attitudes, and practices toward disease self-management.

## Conclusion

In conclusion, patients with diabetes mellitus and hyperuricemia exhibit insufficient knowledge, active attitudes, and poor practices toward disease management. Healthcare providers should prioritize educational interventions targeting these patients, emphasizing the importance of comprehensive disease self-management strategies to improve outcomes.

## Data Availability

The original contributions presented in the study are included in the article/supplementary material, further inquiries can be directed to the corresponding author.

## Footnotes

^1^ http://www.sojump.com
